# Fine needle non-aspiration cytology for the diagnosis of cervical lymph node tuberculosis: a single center experience^☆^

**DOI:** 10.1016/j.bjorl.2018.05.009

**Published:** 2018-06-28

**Authors:** Moncef Sellami, Slim Charfi, Mohamed Amine Chaabouni, Salma Mrabet, Ilhem Charfeddine, Lobna Ayadi, Souha Kallel, Abdelmonem Ghorbel

**Affiliations:** a Habib Bourguiba University Hospital, Department of Otorhinolaryngology-Head and Neck Surgery, Sfax, Tunisia; b Habib Bourguiba University Hospital, Department of Anatomopathology, Sfax, Tunisia

**Keywords:** Cervical, Lymphadenopathy, Cytology, Non-aspiration technique, Tuberculosis, Cervical, Linfadenopatia, Citologia, Técnica não-aspirativa, Tuberculose

## Abstract

**Introduction:**

The fine-needle cytology is being used as a first line of investigation in the diagnosis of head and neck swellings, as it is simple, cost effective and less invasive as compared to biopsy.

**Objective:**

The aims of this study were to evaluate the results of the fine-needle non-aspiration cytology of cervical lymphadenopathy and to study the factors influencing the rate of non-diagnosis results.

**Methods:**

This retrospective study was conducted on selected patients with cervical lymphadenopathy that had undergone a fine-needle non-aspiration cytology followed by a histological biopsy. The sensitivity, specificity, positive predictive value and negative predictive value of fine-needle non-aspiration cytology for diagnosing tuberculosis were estimated. The risk factors of non-diagnosis results were evaluated.

**Results:**

The sensitivity, specificity, positive predictive value rates of fine-needle non-aspiration cytology for tuberculosis were 83.3%, 83.3%, 78.9% and 86.9% respectively. In total, 47 out of the 131 samples (35.8%) were considered non-diagnosis. Of the non-diagnosis samples, 84.2% (38 out of 47) were benign mostly due to tuberculosis (30 cases). Among the studied factors, only tuberculosis (confirmed by histopathological examination) was significantly associated with non-diagnosis cytology (*p* = 0.02, Odds-Ratio = 2.35).

**Conclusion:**

Tuberculosis is currently the commonest cause of cervical lymphadenopathy in North Africa. Fine-needle non-aspiration cytology is safe and accurate in the diagnosis of cervical tuberculous lymph node that is associated with the risk of non-diagnosis cytology.

## Resumo

**Introdução:**

A punção não aspirativa com agulha fina tem sido utilizada como primeira linha de investigação no diagnóstico de tumores de cabeça e pescoço, por ser uma técnica simples, custo-efetiva e menos invasiva quando comparada à biópsia.

**Objetivo:**

Os objetivos deste estudo foram avaliar os resultados de citologia por punção não-aspirativa com agulha fina de linfadenopatias cervicais e estudar os fatores que influenciam a taxa de falha diagnóstica.

**Método:**

Este estudo retrospectivo foi realizado em pacientes selecionados com linfadenopatia cervical submetidos a punção não aspirativa com agulha fina, seguida por biópsia histológica. Foram estimadas a sensibilidade, especiﬁcidade, o valor preditivo positivo e valor preditivo negativo da punção não aspirativa com agulha fina para o diagnóstico de tuberculose. Os fatores de risco dos resultados com falha diagnóstica foram avaliados.

**Resultados:**

As taxas de sensibilidade, especificidade, o valor preditivo positivo e valor preditivo negativo da punção não aspirativa com agulha fina para tuberculose foram de 83,3%, 83,3%, 78,9% e 86,9%, respectivamente. Das 131 amostras, 47 (35,8%) foram consideradas como falha diagnóstica. Das amostras não diagnosticadas, 84,2% (38 de 47) eram benignas, principalmente devido à tuberculose (30 casos). Entre os fatores estudados, apenas a tuberculose (confirmada pelo exame histopatológico) estava significativamente associada à citologia com falha diagnóstica (*p* = 0,02, *odds ratio* = 2,35).

**Conclusão:**

A tuberculose é atualmente a causa mais comum de linfadenopatia cervical no norte da África. A punção não aspirativa com agulha fina é uma técnica segura e precisa no diagnóstico de linfonodos cervicais associados ao risco de citologia com falha diagnóstica.

## Introduction

According to the 2015 World Health Organization report, the prevalence and incidence of Tuberculosis (TB) in Tunisia were 42/100,000 and 33/100,000, respectively.^1^

Lymphadenopathies are the most common form of extrapulmonary tuberculosis and tuberculous lymphadenitis is the most common cause of peripheral lymphadenopathy in a developing country.^2^

The gold-standard procedure for the diagnosis of a cervical lymphadenopathy is open biopsy with histological examination of the excised tissue.^2^

The fine-needle cytology is being used as a first line of investigation in the diagnosis of head and neck swellings, as it is simple, cost effective and less invasive as compared to biopsy.^3^ This procedure has not been commonly developed in North Africa, as most clinicians still use primary surgical excision biopsies.

The objectives of this retrospective study were to evaluate the results of fine-needle non-aspiration cytology (FNNAC) of cervical lymphadenopathy and to study the factors influencing the rate of non-diagnosis (ND) results.

## Methods

The present study was conducted on selected patients presenting an enlarged cervical lymphadenopathy. This study was limited to the selected cases that had undergone a FNNAC from the cervical lymphadenopathy and followed by a subsequent excisional biopsy of the same lymphadenopathy or a biopsy of the suspected primary site for a definitive histopathological diagnosis. Patients with missing FNNAC reports or those cases who could not undergo biopsy were excluded.

In each case detailed history, clinical presentation of cervical lymphadenopathy and clinical examination were carried out.

An ENT surgeon performed the FNNAC. The lesion is immobilized with one hand, and after disinfecting the skin, a 25 gauge needle is introduced into the lymphadenopathy with the other hand. The needle is passed through the lesion in the same way as in Fine-Needle Aspiration Cytology (FNAC), but no suction is applied. The material entering the hub of the needle by capillary action is then expressed onto clean glass slides after attaching an air-filled syringe to it. Multiple smears (3–5) are prepared. The air-dried smears (10 min) are stained with the May-Grünwald-Giemsa (MGG) stain for routine cytodiagnosis.

Cytology reports were categorized into four main results (a) “benign diagnosis with recommendation of follow up”; (b) “Malignant metastatic diagnosis with recommendation of searching for the primary tumors”^3^, ^4^; (c) “Malignant primary lymphoma (non-Hodgkin lymphoma or Hodgkin lymphoma) with recommendation of excision for confirmation and immunophenotyping”; (d) “Inadequate smears or non-diagnosis (ND)” because of scanty/acellular samples.

Suggestive or suspicious cases were considered as positive for malignancy as all these cases were investigated and managed seriously.

Cytomorphologically tuberculous lesions were classified into three groups as described by Das et al.^5^ The cytologic features of tuberculous lesions were grouped under three major cytologic response types as follows:

• Type I – Epithelioid granuloma without necrosis;
• Type II – Epithelioid granuloma with necrosis;
• Type III – Necrosis without epithelioid granuloma.

### Statistics

The data were entered to statistical software (version 20.0, SPSS, IBM Company, Armonk, New York).

For the qualitative variables, the percentage was used as the descriptive index and for the quantitative variables, mean and Standard Deviation (SD) or median and Interquartile Range (ICR) was used.

After ruling out the ND results, the Sensitivity (Se), Specificity (Sp), Positive Predictive Value (PPV) and Negative Predictive Value (NPV) of FNNAC to diagnose tuberculosis were calculated.

ND results were studied according to age, size, location of the node and histological result. The cut-off values for quantitative variables (age, time to the first consultation and size) were calculated by ROC curve analysis.

Whenever required, the values were compared using the Chi-squared test to determine the significance in the difference between the variables. We estimated the Odds Ratio (OR) with 95% Confidence Interval (95% CI) of ND results associated with each risk factor.

A *p*-value <0.05 was used as the level of significance.

## Results

FNNAC was done for 131 patients with palpable lymphadenopathy in the cervical neck region. The main characteristics of the patients are given in Table 1. The commonest site of the involved cervical lymphadenopathy was the upper deep cervical lymph nodes (66.4%) followed by the inferior deep cervical lymph nodes (29%). The median size of the lymphadenopathy was 3 cm (ICR = 2).

**Table 1:** Demographic and clinical characteristics of studied patients.

Characteristics	Patients, n°	Values
*Gender*		
Male	54	41.2
Female	77	58.8

*Age (years)*		35.3 ± 18.5

*Medical history*		
Tuberculosis	1	0.7
Alcoholism	8	6
Tuberculosis in the family	2	1.5
Time to first consultation (months)		2 (5)

*Number of lymph nodes*		
Single	61	46
Multiple	70	54
Size (cm)		3 (2)

Values given as % or mean ± standard deviation or median (interquartile range).

**Figure 1:**
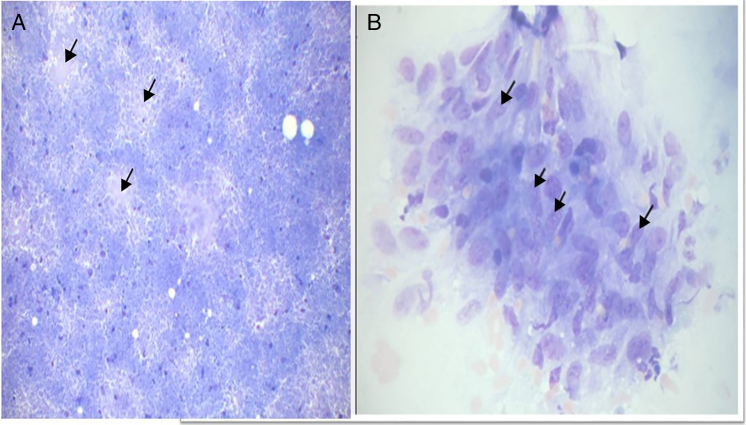
Cytomorphological Type II tuberculosis. A, Caseous necrosis (arrow) and an epithelioid granuloma (MGG ×100).

Table 2 shows the distribution of FNNAC results; 35.8% were benign. Most were tuberculous lymphadenitis (38 out of 47). Among the lymphadenopathy aspirates with tuberculous lesions (39 cases), the Type I, II, and III reactions were observed in 50%, 24% and 26%, respectively (Fig. 1).

**Table 2:** Cytological results of the 131 studied patients.

Cytological diagnosis	No of cases	Percentage
*Benign*	47	35.8
Tuberculous lymphadenitis	38	29.7
Reactive lymph nodes	9	6.8

*Metastatic tumor or suspicious cases*	17	12.9
Metastatic	11	8.3
Suspicious of metastasis	6	4.5

*Lymphoma*	20	15.2
Lymphoma	6	4.5
Suspicious of lymphoma	14	10.7
*Non-diagnosis*	47	35.8

Diagnosis by histology showed; 50.4% tuberculosis, 20.6% reactive lymph node, 17.55% lymphoma and 11.45% secondary metastatic carcinoma.

The cytopathological results were compared with the histopathological diagnoses of the corresponding excised lymphadenopathy or biopsy of the suspected primary site (Table 3).

**Table 3:** Comparative analysis of cytological diagnoses and histopathological diagnoses.

Cytopathological diagnoses	Histopathological diagnoses				Total
	Tuberculosis	Reactive lymph nodes	Metastasis	Lymphoma	
Tuberculosis	30	5	1	2	38
Reactive lymph nodes	1	5	–	3	9
Metastasis	–	1	10	–	11
Suspicious of metastasis	1	3	2		6
Lymphoma	1	–	–	5	6
Suspicious of lymphoma	3	4	–	7	14
Non-diagnosis	30	9	2	6	47
Total	66	27	15	23	131

Data are presented as number of patients.

In total, 84 (64%) samples were considered adequate.

Overall, 70% (59 out of 84) of the adequate samples were in agreement with the histology results for the same patients.

The Se, Sp, PPV and NPV rates of FNNAC for tuberculosis were 83.3%, 83.3%, 78.9% and 86.9% respectively.

In total, 47 out of the 131 samples (35.8%) were considered Non Diagnosis (ND). Of the ND samples, 84.2% (38 out of 47) were benign mostly due to tuberculosis (30 cases).

Based on ROC curve analysis the cut-off values for ND results were an age of 25 years, a time to the first consultation of 7 weeks and a size of the lymphadenopathy of 2 cm.

Among the studied factors, only tuberculosis (confirmed by histopathological examination) was significantly associated with ND cytology (*p* = 0.02, OR = 2.3 and 95% CI = 1.1–4.9) (Table 4).

**Table 4:** Study of risk factor for non-diagnosis cytology.

	Non-diagnosis cytology	Diagnosis cytology	p	Odds-ratio (95% CI)
*Age ≥ 25 years*	31	58	0.7	0.8 (0.4–1.8)
*Age < 25 years*	16	26		

*Time to first consultation*				
<7 weeks	14	36	0.1	1.7 (0.8–3.7)
≥7 weeks	33	48		

*Size of the lymph node*				
< 2 cm	7	16	0.3	0.6 (0.3–1.4)
≥2 cm	40	68		
Superior lymph node	32	55	0.7	0.8 (0.4–1.9)
Inferior lymph node	15	29		

*Diagnosis*				
Tuberculosis +	30	36	0.02	2.3 (1.1–4,9)
Tuberculosis −	17	48		
Metastasis +	2	13	0.053	0.2 (0.05–1.1)
Metastasis −	45	71		
Reactive lymph node +	9	18	0.7	0.8 (0.3–2,1)
Reactive lymph node −	38	66		
Lymphoma +	6	17	0.3	0.6 (0.2–1.7)
Lymphoma −	41	67		

95% CI, 95% Confidence Interval.

## Discussion

Tuberculosis continues to be a major problem of public health interest in North Africa. Our study found tuberculosis to be the commonest cause of cervical lymphadenopathy followed by reactive lymphadenitis and lymphoma.

Lymph node lesions could be found in patients ranging from an early to advanced age.^4^ In our study the youngest patient in the present study was 3 years old and the oldest one was 83 years old.

In 1981, fine needle sampling without aspiration, called as Fine Needle Non-Aspiration Cytology (FNNAC) was introduced.^6^ This technique (non-aspiration) allows better control of the hand during the procedure and a good perception of the consistency of the lesion.^7^

Srikanth et al. compared FNAC and the FNNAC techniques in head and neck swellings and founds that FNNAC technique provides an adequate cellular yield for a definite diagnosis in all head and neck swellings with a statistically significant better retention of architecture in FNNAC smears from lymph node lesions.^6^

Diagnosis of tuberculosis depends upon the demonstration of epithelioid granuloma with or without necrosis. Other reactive components, such as lymphocytes, polymorphs, and Langhan's giant cells, may or may not be present.^5^ Cases in which FNAC smears contain necrotic material without epithelioid granuloma have also been considered as tuberculous lesions, as we do in this study.^8^

In our study, most common cytological pattern of tuberculosis was presence of epithelioid cell granuloma (Type I), which was observed in 50% of cases (19 samples). However, tuberculosis was confirmed in only 12 cases (true positives). FNNAC was found to be a highly accurate method in the diagnosis of tuberculosis; with a sensitivity and specificity of over 80%. This compares favorably with other studies done elsewhere in the developing world where tuberculosis is endemic (Table 5).

**Table 5:** Results of fine-needle aspiration for the diagnosis of tuberculosis.

Diagnosis	Origin	Sensitivity (%)	Specificity (%)	PPV (%)	NPV (%)
Muyanja^9^	Uganda	93.1	100	100	78.9
El Hag^10^	Saudi Arabia	97	100	100	93
Prasad^11^	India	83	94		
Adhikari^2^	Nepal	80	100	100	82
Abdissa^12^	Ethiopia	88.4	48.8	86.1	54.1
Our study	Tunisia	83.3	83.3	78.9	86.9

PPV, Positive Predictive Value; NPV, Negative Predictive Value.

These findings were different from a retrospective, 5 year study from a public hospital in the United States, where FNA was found to have a low sensitivity of 53% in the diagnosis of tuberculosis.^13^ We found six false negatives diagnoses made on FNNAC for the diagnosis of tuberculosis when compared with histology. This is comparable with previous studies.^9^, ^10^

Failure to establish an accurate diagnosis may due to sampling error and in these circumstances, repeat aspiration or excisional biopsy may be considered.^14^ In our experience, the ND rate was 35.8%. The non-diagnostic rate for ND FNA according to the literature ranges from 0.9% to 48%.^9^, ^15^

Rammeh et al. studied the factors influencing the rate of non-diagnosis FNA and found that this rate depends on the size <1 cm, submandibular location of the lymph node, and the experience of the aspirator.^14^

The fibrosis or the extensive necrosis found in tuberculosis may also explain the rate of ND associated with this condition in our study (30 out of 47). Thus, we found that tuberculosis confirmed by histology was significantly associated with a non-diagnosis FNNAC.

The experience of the aspirator is an important factor determining the quality of FNA. Singh et al. investigated 5226 FNAC samples from the six commonest sites and compared the inadequate rates.^16^ The authors observed that the rate of ND were lowest when FNAC was performed by a cytopathologist (12%) and highest when done by a non-cytopathologist (32%).

Ahn D. stated that with training and experience managing at least 100 ultrasound-guided FNAC cases, surgeons can ensure a low inadequate sampling rate and good diagnostic accuracy.^17^

The improved efficiency of ultrasound-guided FNAC over palpation-guided FNAC in the head and neck masses has been well documented, leading to its acceptance as the standard of care among radiologists and many cytopathologists.^18^, ^19^ The addition of ultrasound guidance reduces the non-diagnostic rate.^20^ It is however a more expensive technique than non-ultrasound guided FNA and should be performed for lymphadenopathy that are small in size or in difficult locations.^20^ In our study, we used the palpation-guided technique in all cases.

Repeating cytology is useful and should be considered especially in the case of non-diagnosis cases. In the study of Shykhon et al., ND was 48% in the first cytology and dropped to 32% after the second.^15^

## Conclusion

In summary, our study shows that tuberculosis is currently the commonest cause of cervical lymphadenopathy in North Africa. This condition was significantly associated with ND cytology

FNNAC is safe and accurate in the diagnosis of cervical tuberculous lymph node that is associated with risk of non-diagnosis cytology.

## Conflicts of interest

The authors declare no conflicts of interest.
